# Beaver-generated disturbance extends beyond active dam sites to enhance stream morphodynamics and riparian plant recruitment

**DOI:** 10.1038/s41598-019-44381-2

**Published:** 2019-05-31

**Authors:** Rebekah Levine, Grant A. Meyer

**Affiliations:** 1 Department of Earth and Planetary Sciences, University of New Mexico, MSCO3-2040, 1 University of New Mexico, Albuquerque, New Mexico 87131 USA; 2 Environmental Sciences Department, University of Montana Western, 710 S. Atlantic St., Dillon, Montana 59725 USA

**Keywords:** Riparian ecology, Freshwater ecology, Geomorphology

## Abstract

Given the direct effects of their dams on hydrology, sediment storage, and vegetation, beaver are widely acknowledged as ecosystem engineers. Here we explore the effects of beaver activity on channel processes and riparian plant recruitment beyond those dams and after dam abandonment in southwestern Montana, USA. Willow cuttings from beaver herbivory are commonly deposited along point bars, adding roughness and promoting sediment accumulation. Most cuttings are found <1 km downstream of an active dam. These cuttings often sprout, aiding in willow colonization and bar stabilization. Thirty-four radiocarbon ages show that beaver cuttings have accumulated by similar processes over thousands of years, adding to floodplain carbon storage. Breached dams can initiate meanders, increasing channel and riparian habitat diversity. Beaver activity thus generates a cycle of frequent disturbance, from dam building and riparian plant browsing through dam failure and abandonment, with each phase influencing channel and floodplain evolution and riparian plant recruitment.

## Introduction

Riparian ecosystems are coming under increasing stress from changing climate, increasing demands on water, and loss of flood-induced disturbance^1^. In addition, given the strong influence of beaver on fluvial and riparian function, and their decline across much of their native Northern Hemisphere habitat^2^, these ecosystem engineers have become an important area of focus in riparian science. Attention has centered on the effects of intact dams^3,4^ and direct impacts of herbivory^5,6^. Nonetheless, there has been limited investigation of the effects of beaver activity on stream reaches and riparian vegetation beyond their iconic dams and ponds, or on the continuing impacts of breached and abandoned dams. For example, beaver are notorious herbivores, generating a steady supply of beaver-chewed wood^7^ that is often transported downstream. Although seed dispersal may be the dominant and critical form of reproduction for riparian plants, sprouting from stem fragments can also play an important role in riparian plant reproduction, particularly for species that are adapted to floodplain disturbance^8^. In prior research along beaver-occupied streams in southwestern Montana and the Rocky Mountain region, we observed that willow cuttings produced by beaver herbivory and dam building are commonly transported downstream and accumulate along channel margins. Thus, we asked whether beaver-generated willow cuttings are deposited and sprout in sufficient quantities to facilitate riparian colonization in reaches below active beaver dams; whether cuttings and sprouts promote further sediment deposition; and if cuttings deposition has been a widespread and long-continued process, which can be addressed through identification and radiocarbon dating of subfossil willow cutting accumulations. In addition, we saw that beaver dam remnants often persisted for some years after breaching and abandonment, with the potential to influence channel morphology and evolution. Here, we document the continuing effects of remnant dams to consider the impacts of beaver beyond the life of active dams and ponds.

**Figure 1:**
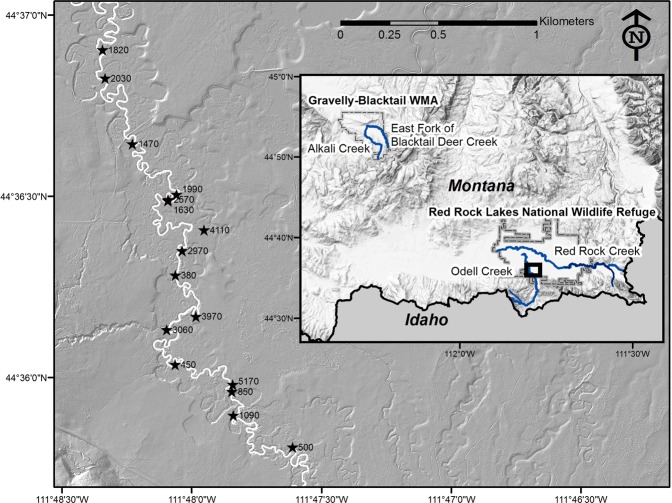
Inset map (ESRI ArcGIS Online, © 2007 National Geographic Society, i-cubed) shows the study streams in blue with bold box showing location of main map. Main lidar shaded relief map (doi.org/10.5069/G9NP22C7) shows sites with beaver cutting deposits along Odell Creek. Radiocarbon ages for the oldest beaver cuttings sampled at each site are shown as the median of the calibrated age probability distribution^57^, rounded to the nearest 10 years; uncertainties and measured radiocarbon ages can be found at: 10.17605/OSF.IO/B2JWS.

The entire network of a beaver occupied stream appears to be influenced by their presence, as dam sites cycle through being maintained, abandoned and ultimately failing. We have focused our work on three streams in the upper headwaters of the Missouri River System in southwestern Montana (Table 1, Fig. 1) that all feature unconfined meandering alluvial channels^9^, where active beaver colonies exist within protected landscapes with limited human impacts. On the three study streams, we document and quantify beaver cutting dispersal and additional less-direct effects of beaver activity within the dam-building and abandonment cycle, including interactions with channel processes.

**Table 1:** Characteristics of study streams.

Stream	Channel Type	Mean Slope (m/m)*	Basin Area (km²)^	Mean Annual Q (m³/s)	Mean Peak Q (m³/s)	Mean Reach Sinuosity*	Data Citations
Odell Creek	gravel bed, pool-riffle, meandering channel	0.004	45	1.32 (1.5 in 1998)	10.01	2.9	USGS 06008000 Odell Cr ab Taft Ranch nr Lakeview MT, (1994–1998, accessed 5 March 2019)
Red Rock Creek	gravel bed, pool-riffle, meandering channel	0.003	97	1.35 (2.07 in 1998)	4.62	2.1	USGS 06006000 Red Rock Cr ab Lakes nr Lakeview MT, (1998–2018, accessed 5 March 2019)
East Fork of Blacktail Deer Creek (EFBDC)	gravel bed, pool-riffle, meandering channel	0.009	125	*0.85* ^*#*^	*6.62* ^*#*^	1.8	Discharge estimates from USGS StreamStats^59^ (accessed 5 March 2019)
Alkali Creek	gravel bed, plane bed, limited meandering, narrow floodplain	0.016	20	*0.1* ^*##*^	*1.42* ^*##*^	1.4	Discharge estimates from USGS StreamStats^59^ (accessed 5 March 2019)

^*^Calculated for study reaches.

^^^Area contributing to flows at flow measurement locations from within study reaches, Calculated from USGS StreamStats, 5 March 2019.

^#^Data are not measured, but estimated from regression equations.

^##^Estimated discharge calculations are problematic at small basin area sizes, although these data have been corroborated with measured discharges and give a reasonable estimate of annual flows.

## Results

### Accumulations of willow cuttings from beaver herbivory

**Figure 2:**
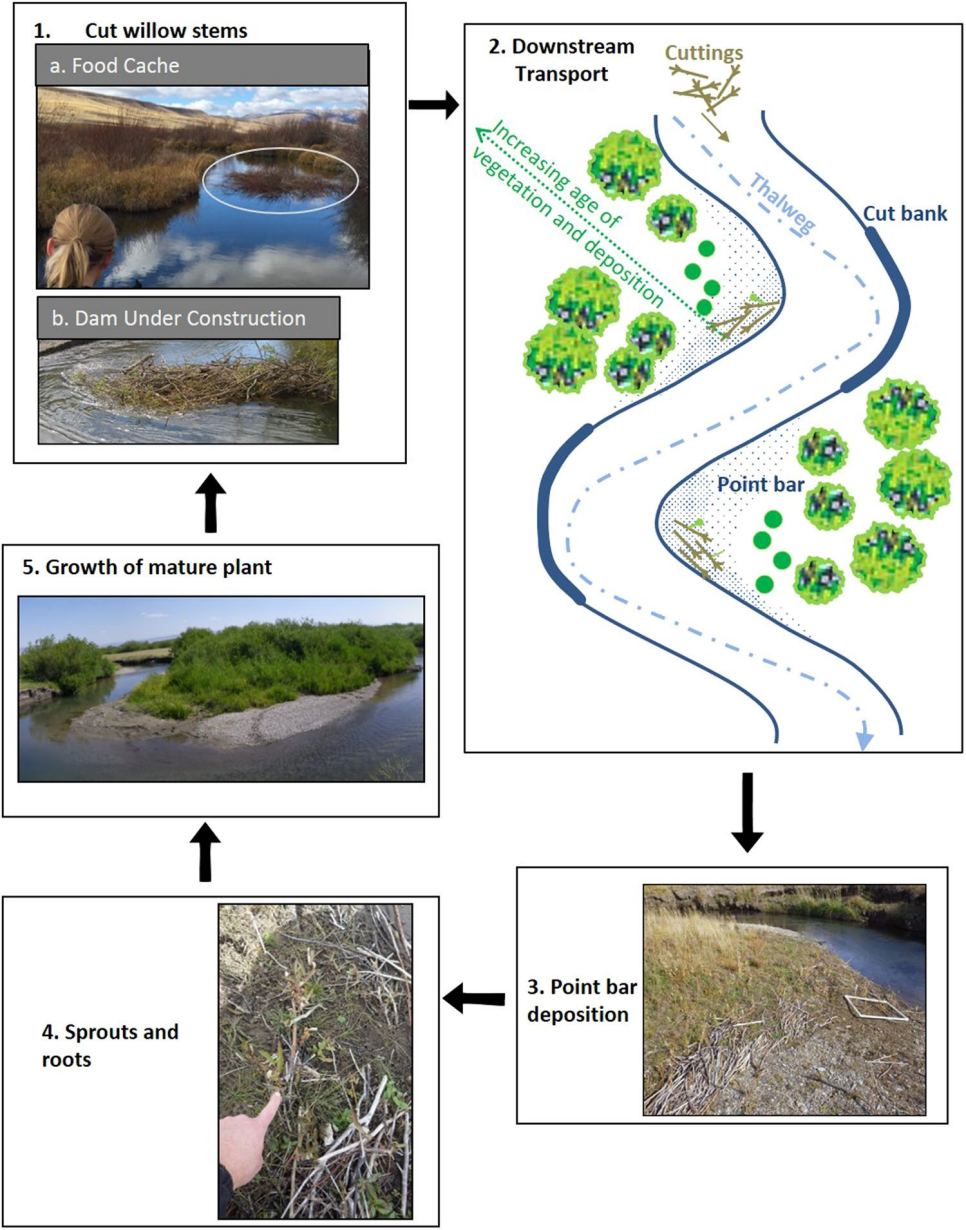
Beaver generation of willow cuttings (plant propagules) and distribution. **1.** Willow food cache on East Fork Blacktail Deer Creek (**1a**); beaver cut willow for dams on Odell Creek (**1b**) **2.** Some cuttings from loss or dam breaching are transported downstream. The dashed line shows the thalweg, i.e. the deepest part of the channel. The cuttings are eventually deposited in areas of low velocity and shear stress, often on the downstream margin of point bars, along with fine sediment and willow seeds. **3.** The cuttings add roughness contributing to further fine sediment accumulation. **4.** Some of the cuttings develop adventitious roots and sprout on the wet substrate of bars. **5.** Sprouted willows grow into mature plants, increasing roughness and promoting point-bar growth and meander development. All willows in this Odell Creek photograph are growing on point bars, whereas the cutbank in the background is formed in a higher fluvial terrace with grassy vegetation. The youngest willows lie near the toe of the bar, coincident with the youngest sediment (see also (2) in this figure).

**Figure 3:**
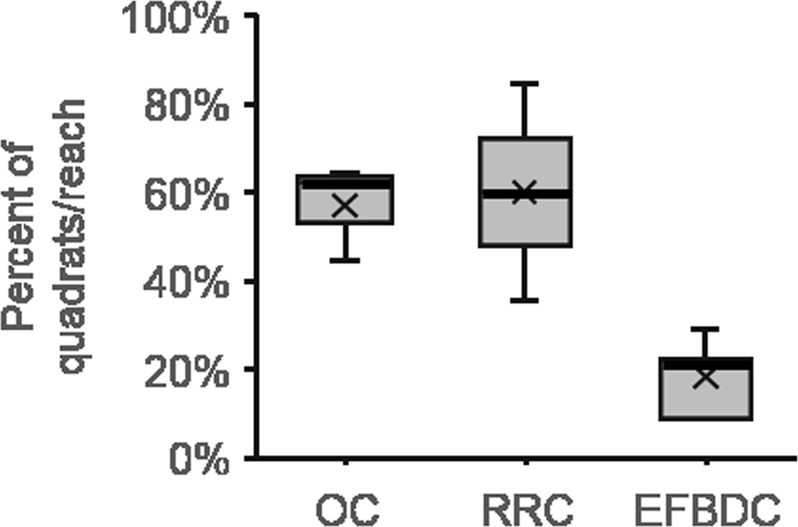
Box plot showing percent of 0.5 × 0.5 m quadrats within each reach (45 quadrats per reach), that contain willow cuttings produced by beaver herbivory along Odell Creek (OC, mean gradient = 0.004 m/m, mean sinuosity = 2.5, 3 reaches), Red Rock Creek (RRC, mean gradient = 0.003 m/m, mean sinuosity = 2.2, 3 reaches) and East Fork of Blacktail Deer Creek (EFBDC, mean gradient = 0.008 m/m, mean sinuosity = 1.8, 5 reaches), Beaverhead County, Montana. Thick lines show medians; x symbols indicate means; box limits denote 25^th^ and 75^th^ percentiles; whiskers extend 1.5 times the interquartile ranges; there are no outliers; medians are included in calculations of quartiles.

The feeding activities of beaver and the construction, maintenance, and breaching of beaver dams provide a steady supply of fine woody debris to the channel. Beaver-cut willow stems (hereafter termed cuttings) are generally small in diameter (1–3 cm), with a mean length of 10.4 cm (SE ± 0.22). The cuttings are deposited in clusters along the margins of the study streams and cover large areas of the point bars (Figs 2, 3). At 90 sites along Odell Creek, we recorded a summed total length of 228 m of beaver cuttings within 270 0.5 × 0.5 m quadrats. Beaver cuttings accumulated on 81% of point bar sites and 51% of all surveyed sites along Odell Creek, demonstrating the ubiquitous character of this process. Red Rock Creek and East Fork of Blacktail Deer Creek (EFBD Creek) showed similar accumulations of cuttings on point bars in meandering reaches (Fig. 3).

### Controls on beaver cutting accumulations

To understand controls on beaver-cutting deposition, we investigated variability in accumulation along the margins of Odell Creek, Red Rock Creek and EFBD Creek. Below, we report the results of linear mixed effects analyses^10^, where Chi-squared likelihood ratio tests were used to assess how each parameter affected mean willow cutting length at sites as: χ^2^ (degrees of freedom) = test statistic, p = p-value. Where appropriate, we also include the mean and standard error of the mean (SE).

**Figure 4:**
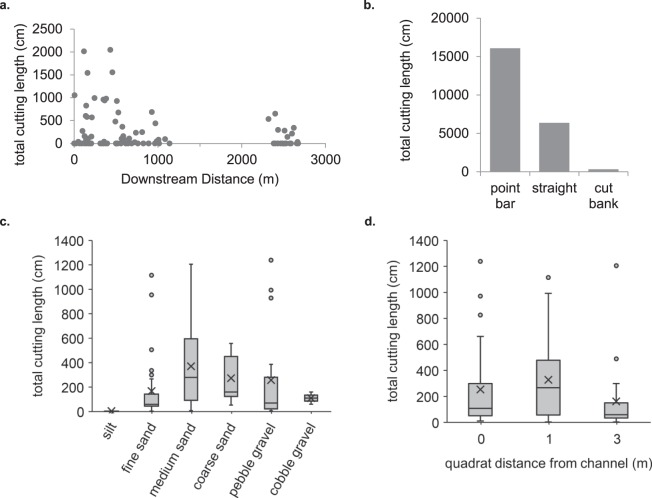
Summed length of willow cuttings produced by beaver herbivory relative to potential controlling factors along Odell Creek, Montana. (**a**) Summed cutting lengths by site plotted versus site distance to nearest upstream dam. All sites are shown. (**b**) Summed cutting lengths for each channel morphological class across all sites. (**c**) Box plots showing summed cutting length across 0.5 × 0.5 m quadrats in each sediment class; center lines show medians; x symbols indicate means; box limits denote 25^th^ and 75^th^ percentiles; whiskers extend 1.5 times the interquartile ranges; outliers are represented by points; medians are included in calculations of quartiles. (**d**) Box plots showing summed cutting length across quadrats in each distance class (0, 1 and 3 m), where distances are from low-flow water edge. Box symbols and calculations are the same as in c.

As beaver harvest willow for dam construction, food caches, and immediate consumption near active dam sites, these sites were hypothesized to be the most important parameter influencing total willow cutting length. Model results show that the distance downstream from an active dam significantly explains variability in total cutting length (χ^2^ (1) = 4.49, p = 0.03) (Fig. 4), and that total cutting length decreases by 0.06 cm/m (SE ± 0.03 cm/m) downstream of a dam site. We also hypothesized that the total number of dams upstream of a site would be important in the total cutting length, but this was not the case, as the farthest downstream site, with 7 dams on the stream above it, had the lowest total cutting length. Even the most upstream reach, with only 2 dams above it, had a greater total cutting length. Most cuttings are found less than ~1 km downstream of an active dam. These findings suggest that the mean travel distance of cuttings between sources and depositional sites is relatively low, and that dams efficiently trap cuttings from upstream.

Channel morphology strongly controls deposition of willow cuttings, creating distinct populations of cutting accumulations among point bars, straight reaches, and cutbanks (χ^2^ (2) = 6.41, p = 0.04), where point bars accumulate the greatest cutting length (mean = 202.8 cm (SE ± 33.2)), and cutbanks the least (Fig. 4). Because point bars were dominant locations of accumulation, we subdivided point bar sampling sites by upstream, midpoint and downstream locations. Point bar location was important in explaining much of the variability in total cutting length between sites (χ^2^ (2) = 770.13, p < 0.001), with downstream bar sites having the greatest cutting length (mean = 278.9 cm (SE ± 59.1 cm)), followed by upstream bar (mean = 235.4 cm (SE ± 65.1 cm)) and midpoint bar sites (mean = 126.7 cm (SE ± 54.9 cm)). Throughout a bend, the point of maximum shear stress moves from inside to outside of the bend. Greater cutting accumulation on the downstream edges of bars is consistent with low boundary shear stress in meander-bend flow^11^.

Without directly measuring shear stress, sediment size provides a way to understand the relative shear stress of cutting accumulation sites. The expected variations in shear stress around meander bends is reflected in grain size distributions, where the coarsest sediments are found in pools near the outside bank, just downstream of the point bar, while fine sediments are transported toward the inside and downstream part of the bend, where shear stresses are lower^11^. Sites with lower shear stress are sites that may protect seedlings from scouring flows. Grain size distributions are not only important for understanding shear stress, but also for determining whether cuttings are being deposited in sandy sediment that retains moisture needed for willow establishment^12^. Fine and medium sand were most common at downstream point bar quadrats at 1 m from the low-flow channel (n = 10 and 11 respectively) and were associated with large cutting accumulations (Fig. 4), although medium sand quadrats contained the greatest cutting accumulations across all distances from the channel (Fig. 4).

Location relative to the active channel explains a significant amount of the variability in total length of cuttings between quadrats (χ^2^ (2) = 618.6, p < 0.001) (Fig. 4). Within each site, quadrats were placed at the low-flow stream edge and at 1 m and 3 m from the stream edge. The 1 m quadrats accumulated the greatest total length (mean = 323.8 cm (SE ± 55.62 cm)), followed by the quadrats adjacent to the low flow channel (0 m quadrats, mean = 247.3 cm (SE ± 51.1 cm)). The 3 m quadrats were the least likely to accumulate cuttings (mean = 164.2 cm (SE ± 70.9 cm)). In most cases, sites at the 3 m distance were on average 70 cm above the low-flow channel elevation and 40 cm above the bankfull channel elevation, and only larger floods are likely to push cuttings this far onto the floodplain.

Channel sinuosity, morphology and hydraulics appears to control cutting accumulation as well. Along Odell Creek, the upper reaches with steeper gradient and coarse gravelly bed sediment have no major accumulations of beaver cuttings. Field observations also indicate that the lower gradient, meandering reaches of Odell Creek and Red Rock Creek accumulate more willow cuttings than the higher gradient, less sinuous reaches of EFBD Creek (Table 1, Fig. 3). On small headwater streams such as Alkali Creek (Table 1), the lack of well-developed point bars appears to inhibit beaver cutting accumulations, even though consecutive beaver occupancy surveys indicate that dams may persist there for at least a decade. On larger regional rivers such as the Madison and Clark Fork Rivers in Montana^13^, beaver have currently and historically occupied smaller side channels; these exhibit some beaver cutting accumulations, but the cuttings appear to have limited influence on river processes in these high-energy systems.

### Beaver cuttings and willow recruitment

We counted sprouts from cuttings in each quadrat, which yielded a mean number of sprouts per quadrat of 0.5 (SE ± 0.1), with a total of 72 sprouts across all quadrats in the study area. Although this number is relatively low compared to the total length of cuttings, sprouts were present at 25% of all sites and appeared to be more numerous with a greater total cutting length (Fig. 4). Sprouts were most commonly associated with sites with sandy deposits, especially medium sand. Forty-five percent of medium sand quadrats had sprouts, with a mean of 1.2 sprouts per quadrat (SE ± 0.4), possibly because of significantly more cutting length available for sprouting (7620 cm). Coarse sand quadrats had the highest mean sprout content (1.6 sprouts/quadrat (SE ± 1.4) and showed the greatest sprouting success per available cutting length (1020 cm). Overall, these data indicate that beaver herbivory results in deposition of abundant cuttings on suitable substrates, making vegetative propagation a viable mechanism for recruitment. We tested whether total cutting length at a site had a strong control on the number of sprouts produced and found that the relationship was not significant.

### The effect of beaver cuttings on channel processes, floodplain evolution and carbon burial

Cuttings added to stream channels by beaver activity are also likely to alter sedimentation processes at low-energy depositional sites. Although the cuttings are small in diameter, they are deposited in clusters (Fig. 2) with the initial deposition of fine woody debris making it more probable that additional cuttings will be trapped at the site^14^.

**Figure 5:**
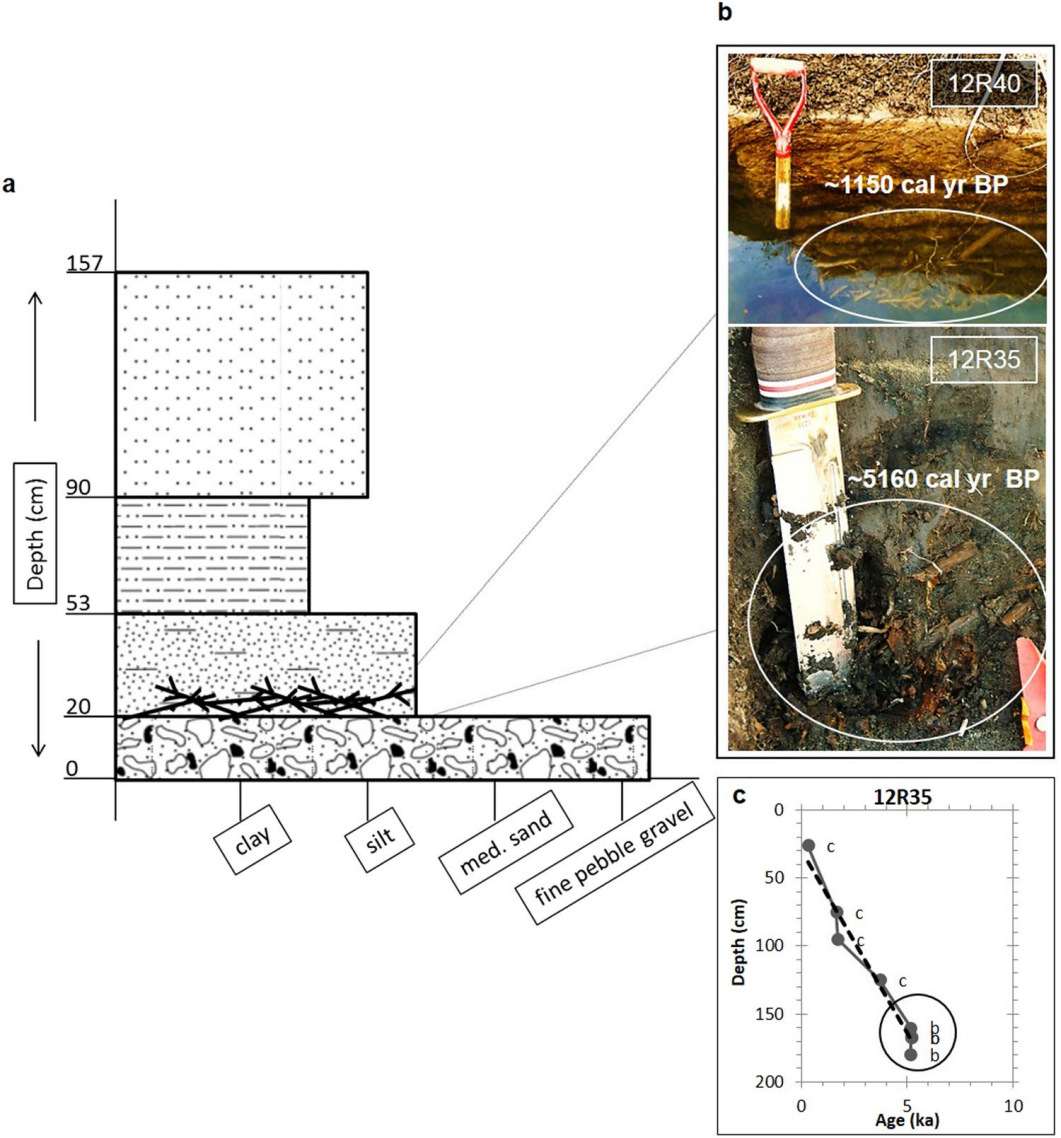
(**a**) A composite stratigraphic column showing the typical context of beaver willow cuttings buried in Holocene sediments along Odell Creek. Units observed at most sites and their mean thickness and grain size are shown. Cartoon sticks in the second unit from the bottom show the typical position of cutting deposits. (**b**) Photographs show examples of *in situ* cuttings at 2 sites, where the top photo shows cuttings exposed below the water surface; median calibrated radiocarbon ages^57^ for collected samples, rounded to the nearest 10 years, are noted. White ellipses highlight the cuttings in each image. (**c**) Plot shows median calibrated radiocarbon ages and depths of collection for site 12R35 – also shown in lower photograph in (**b**); “c” represents charcoal and “b” represents beaver cutting samples. The circle highlights the beaver cutting ages and depths.

The abundance of beaver cuttings in older Holocene-age deposits along Odell Creek and Red Rock Creek demonstrates that beaver cuttings accumulating on point bars is ubiquitous and has been throughout the Holocene. Beaver cuttings, preserved for millennia, are evidence of relatively rapid burial and demonstrate that beaver-inhabited headwater alluvial streams are sites for long-term storage of detrital organic matter (Figs 1, 2, 5)^9^. Holocene terraces from 1.2–3 m above the low-flow channel represent former floodplains of these streams, and their deposits are commonly well-exposed in cutbanks and well-distributed throughout the meandering middle reaches of the creeks. Thirty-four radiocarbon (^14^C) ages show that beaver cuttings range in age from ~6030–380 cal yr BP (Fig. 1) demonstrating that deposition of beaver cuttings has been a common process over millennia. The subfossil beaver cuttings appear in discrete, laterally extensive layers with high concentrations of willow stems, cleanly cut at an angle typical of beaver-chewed wood (Fig. 5). The cuttings layers have a narrow range in radiocarbon age and are mostly contained within sandy sediment overlying coarser sand, pebbles, or gravel; on top of the cuttings are fine sands grading to thick deposits of sandy silt. This upward-fining trend is typical of point-bar deposits and overlying overbank sediments^15^. Sedimentary sequences associated with abandoned beaver dams in the study region feature localized woody debris accumulations, berm-like surface forms, and thick, muddy, organic-rich pond sediments^16^. In contrast, we find that Holocene sediments containing beaver cuttings along the meandering streams of this study most closely resemble point-bar deposits. The abundance of beaver cuttings on modern point bars (Fig. 4(b)), including those being buried by sediment, provides a strong modern analog in support of this interpretation.

### Active versus abandoned dams

Beaver dams in southwest Montana streams have variable longevity as intact structures, but the breached remnants of those dams persist much longer and make up the majority of instream beaver structures. In the field, abandoned dams were identified where some remnant of the dam lay within the main channel. In summer 2009, 10 km of Odell Creek contained 9 active dams and 21 abandoned dams. In surveys of 10 km of EFBD Creek in 2015, 24 active dams and 33 abandoned dams were identified. On both Odell Creek and EFBD Creek, long-term monitoring shows that beaver reoccupy sites at >5 year intervals, with locations of beaver occupancy shifting throughout the available habitat along the streams. Dam sites are active over an average of three years on these two midsize streams. We measured the upstream influence on water surface slope on Odell Creek and EFBD Creek and found that active dams are affecting slope over 3% and 4%, respectively, of the total surveyed channel length. On smaller, adjacent Alkali Creek, a 2016 survey identified 24 active dams and 24 abandoned dam sites in 10 km, and comparison with a 2009 survey and USDA NAIP imagery from 1995–2014 show that dam positions for 6 of the largest dams have remained consistent for 7 years. One of the large beaver wetlands on Alkali Creek has remained intact since 2005, with others persisting for >5 years. On Alkali Creek, abandoned dam sites maintained an average of 70% of cross-channel extent. On Odell Creek and EFBD Creek, some dam remnants can persist within channels and influence flow patterns and sedimentation for at least a decade.

Along the study steams, we observed that meanders had been initiated by beaver dam remnants that deflected flow toward the opposite bank^17^. Dam remnants that were stabilized by live plant stems sprouted from cut willows used in dam construction were particularly effective in directing flow. In typical meander bends, shear stress is concentrated at the outer bank, promoting cutbank erosion and the transport of coarse bed load, whereas fine sediment is deposited along the inner bank^11^. In the case of a breached dam, a remnant can act as the inner bank, with deposition further enhanced by sprouting willow. Thus, the beaver dam remnant forces the channel to shift laterally over time.

As beaver occupy and abandon sites, dam sites shift from intact to breached, to abandoned, creating a mosaic of habitats that influences reproductive opportunities for riparian plants and aquatic organisms. Dams along our study streams elevate water levels within the channel and enhance wetlands across the floodplain. The 8 largest wetlands on EFBD Creek total 4.6 hectares over 4 kilometers of stream, while total beaver-generated wetlands on adjacent Alkali Creek total 1.2 hectares over 10 kilometers of stream. All wetlands were delineated during the baseflow period when flooding would not otherwise be occurring.

Intact dams can also enhance channel dynamics by promoting avulsions. In ten years of observations on Odell Creek, two channel avulsions were associated with beaver damming. Each avulsion caused abandonment of a channel segment, resulting in sediment trapping within the segment and initiation of sediment stabilization as vegetation began developing on bars (Supplementary Fig. 1). One of the avulsions created a new wetland adjacent to the redirected channel. We observed additional beaver dams producing overbank flow during seasons and low-flow years when flooding would not otherwise be occurring. Raising local flow stage creates a patchwork of disturbance environments, affecting riparian plant communities through inundation die-offs, and initiating primary succession on bare mineral substrate left behind by receding floodwaters, as documented in other studies^18^.

## Discussion

In all states of repair, beaver dams induce disturbance that enhances stream dynamics and riparian ecosystem function. Active, intact dams have been recognized as beneficial to restoring incised streams with disconnected floodplains^19,20^, and for some time have been seen as an important tool in stream restoration^21^. Our observations provide additional support for the impacts of intact dams. At any point in time, however, active beaver dams affect a small proportion (<5%) of the total channel length on our Montana study streams. Breached or failing beaver dams nonetheless have persistent effects on fluvial processes and can produce desirable restoration outcomes^17,22,23^. Breaches, where part of the dam remains intact, are common within our study sites, and on other North American streams^24^. Dams usually breach under high stress during flood discharges^24^, typically during the snowmelt pulse on the Montana study streams^17^.

Like large woody debris^25,26^, the remnants of breached beaver dams perturb water surface slope and add channel roughness, especially where cuttings have sprouted^27^. Both the decrease in slope and the added roughness promote sediment retention, raising bed elevations near the dam and creating a topographic high that begins to act like a point bar^17^. In the field of river restoration, a project is usually considered a failure when constructed elements “blow out”^28^. In the case of beaver dams, however, the remnants of a “blow out” continue to promote habitat diversity beyond the effects of the initial structure. Dam breaching is a critical part of the beaver cycle in streams and acts as an additional disturbance in the riparian corridor, an outcome desired in many restoration projects^29^.

The addition of small-diameter willow cuttings may act in a similar way to dam remnants, though the effect is less direct. Hydraulic roughness is increased by their accumulation, and as additional cuttings are trapped and roots are established, the effect will increase. Small roughness elements—including larger gravel^30^, grass, and willow sprouts—can stabilize bar sediments and promote additional sedimentation^31^. In developing meanders, inner-bank deposition is the predominant process during frequent, small floods^32^. As the point bar builds outward, aided by roughness from willow cuttings, more flow is directed toward the outer cutbank^33^ and shear stress is reduced on the inside of the bend.

More broadly, the cycling of beaver activity through the channel network promotes habitat heterogeneity and enhances reproductive opportunities for riparian plants. At any given time, a typical functioning beaver stream encompasses a mosaic of site types, including intact dams, recently breached or abandoned dams, and long-abandoned dams, often interspersed with reaches unsuitable for beaver colonization^34^. Beaver relocate when resources are depleted at their dam site and move to where preferred food is available^35^. Some sites can sustain a beaver colony for many years, while some may be occupied for only a brief interval^2^. Site occupancy also depends on suitable geomorphic characteristics of a particular site, which varies between reaches and catchments^16,17,34^.

Shifting occupancy by beaver creates ever-changing areas of fresh, moist sediment, including some where vegetation is also recovering following a die-off from inundation^27,36^. For riparian vegetation, the shifting mosaic of beaver activity can generate variability in ages and species distributions^2,6,36^ that also results in spatial variations in overbank flow on floodplains. For example, with their closely spaced stems and dense low canopies, willows promote deposition, and offer more protection from erosion in extreme flood flows than cottonwoods^27^. Riparian communities along streams that lack beaver or large wood are dependent on flood disturbance and channel migration to provide colonization sites on bare sediment^37,38^, thus shrub and tree distribution is closely tied to flood occurrence. Flood levels must also be fortuitously timed with seed dispersal^39^. The result is vegetation in cohorts with ages tied to flood events^38^. In beaver-occupied stream systems, however, beaver produce a steady supply of cuttings and substrates for regeneration (Figs 2, 4), allowing plant recruitment to occur throughout the growing season^40^. Beaver are most active during the summer months. Declining discharges could strand beaver-cut stems over a range of elevations below crest stage, just as seeds are stranded by falling flows^41^. The data indicate that moderate to low flows deposit most of the cuttings, coincident with times when beaver are most actively harvesting willow during declining discharge after snowmelt runoff peaks in late May and early June.

**Figure 6:**
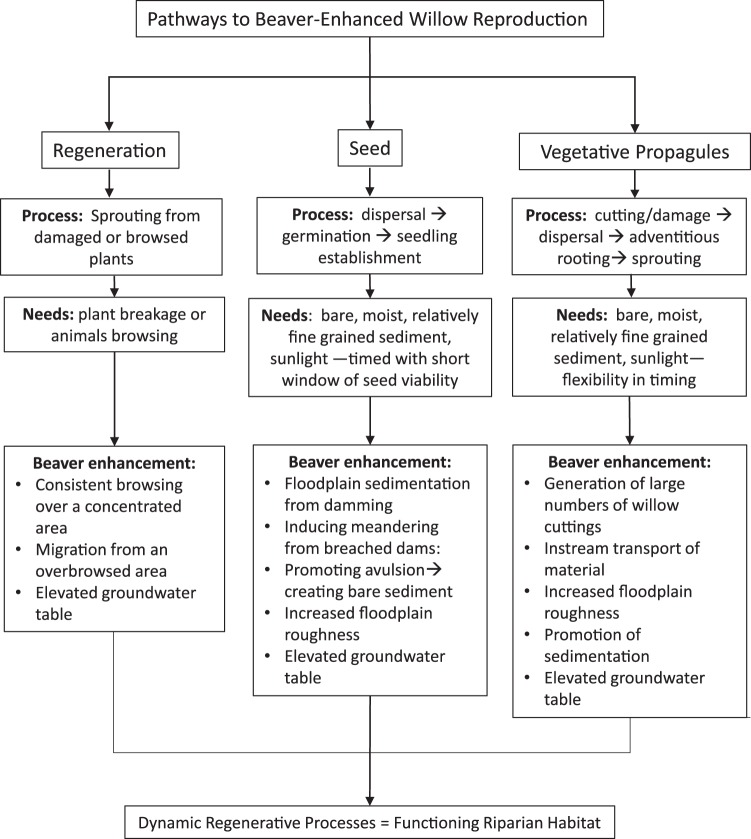
Overview of beaver enhancement of willow reproduction through regeneration from the parent plant (asexual reproduction), seed (sexual reproduction) and vegetative propagules (asexual reproduction from plant pieces that have broken off the parent plant). Each process is briefly summarized, basic requirements for the processes (needs) are outlined, and the beaver enhancements are listed.

Our work also shows that there is some variability in the degree to which beavers disturb a channel that is related, in part, to stream discharge and sinuosity. Frequent breaching, and therefore the ability of breached dams to affect channel morphology, is more common in streams with greater discharge^17^, while the point bars of more sinuous streams provide sites for establishment of seedlings during frequent bankfull flood events^42^. Consistent with our findings, the downstream sides of point bars were important sites for establishment of willow vegetative propagules following a large flood that occurred out of phase with willow seed release^40^. Additionally, lateral accretion of material in migrating bends provides increasing shelter from scour for saplings^42,43^ Overall, despite beaver impacts being partially dependent on catchment characteristics, the variability in beaver occupancy in both space and time creates riparian patchiness and habitat heterogeneity along the stream corridor^17,34,44^ with beaver enhancing multiple pathways for willow reproduction (Fig. 6).

In addition to reproduction through seeds, which is likely dominant, beaver herbivory and construction of dams and lodges provide a secondary pathway for willow reproduction through the generation of abundant cuttings. Our data show that beaver cuttings are a prolific source of plant propagules to stream networks. Prior work on willow reproduction indicates that floodplain willow cuttings successfully reproduce under a wide range of moisture, sediment and burial regimes—particularly in comparison to non-floodplain willow^45^—supporting vegetative propagules as a viable reproductive pathway in dynamic floodplain environments^46^. Genetic data show that sexual reproduction likely dominates in Salicaceae, but the ratio of asexual and sexual reproduction can vary greatly even between different populations of the same species, with variability attributable to local site factors^46^ – which could include the presence of beaver. Relative to seeds, willow vegetative propagules have a greater reserve of carbohydrates and water within the stem, so are more viable under adverse conditions^8,47^. Without generation of propagules, willows depend on the large quantities of short-lived, non-dormant seeds produced each year^7,48^. Generation of seedlings is highly dependent on newly deposited moist sediment, which in the absence of beaver may occupy a very small percentage of the landscape^18^, and may not be available during the short window of seed viability^40^. Additionally, seeds of willow appear to have a limited range of dispersal^18^, whereas vegetative propagules can float downstream for comparatively long distances^49^, despite trapping by beaver dams and stranding on point bars and other depositional sites along the way. The reproductive strategies of willow along with our findings from Montana streams lead us to hypothesize that the vegetative propagule mode of reproduction should increase in relation to the abundance of beaver in a stream, at least where population size is not strongly limited by food resources, but this remains to be tested.

Our observations of large cutting accumulations on point bars of many other streams in western Montana (e.g., Upper Ruby River, Beaverhead River and tributaries, and Upper Clark Fork River) and across the Rocky Mountain region indicate that fine woody debris accumulations from beaver herbivory are common. In addition, ^14^C dating of these accumulations in Holocene floodplain sediments along Odell Creek and in the greater Yellowstone region^16,50^ shows that cuttings are stored over timescales of millennia (Figs 1, 5), showing that an active beaver dam cycle in headwater streams can both affect channel morphodynamics and promote the long-term burial of organic carbon within floodplain sediments. Headwater alluvial streams with actively migrating channels and beaver activity are important locales for carbon storage in mountain rivers, accounting for ~75% of total carbon in the stream network^9^. Wohl^51^ estimated that wet beaver meadows can account for 23% of the total carbon stored in the landscape, but this may be a conservative estimate of beaver contributions to carbon storage, as beaver-cut stems in point-bar deposits were not included. Along unconfined meadow reaches of mountain streams where large wood is rare, beaver activity may be the predominant mechanism for carbon storage. The multi-millennial preservation of beaver-chewed wood in point-bar sequences along Odell Creek attests to the importance of beavers in carbon storage well beyond dam sites.

Without beaver, initiation of primary or secondary succession requires flood events or channel migration^38^. The loss of beaver from stream systems across northern latitudes has meant that much of the research on functioning riparian systems, riparian plant establishment, and the interplay of water and sediment in streams, is grounded in research from sites lacking beaver^2^. Our work shows, however, that beaver can facilitate multiple pathways for plant colonization (Figs 2, 6) and add to both channel and riparian habitat diversity even after dams are abandoned. Riparian plants reproduce through seed, vegetative propagules and regeneration from damaged plant material, with each pathway augmented by, or predicated on, disturbance. Like other types of disturbance, beaver activity resets succession, allowing pioneer species to gain a foothold. In North American and Eurasia, the full cycle of beaver activity – including dam building, breaching and herbivory – enhances fluvial and floodplain dynamics, and promotes the reproduction and regeneration of riparian plants, not only at active dam sites, but throughout intervening stream reaches as well. Evaluating beaver activity for system-wide effects should be considered in the research and management of fluvial and riparian system dynamics.

## Methods

### Study area

Investigations focused on Odell Creek, with ancillary data on three other streams, East Fork of Blacktail Deer Creek (EFBD Creek), Red Rock Creek and Alkali Creek. All study streams are within the upper headwaters of the Missouri River system in southwestern Montana, USA (Fig. 1). The study reaches on Odell Creek and Red Rock Creek are located within Red Rock Lakes National Wildlife Refuge, and EFBD Creek and Alkali Creek study reaches are located within the Gravelly-Blacktail Wildlife Management Area, managed by Montana Department of Fish, Wildlife and Parks. All study reaches have experienced limited human impact, so provide a valuable reference for studying intact abiotic-biotic interactions. Annual hydrographs across the region are dominated by snowmelt, with peak flows occurring in May and June. The three large streams feature gravel-bed, pool-riffle meandering channels and broad floodplains over most of the study reaches; Alkali Creek shows low-sinuosity meandering in some sections, but is predominantly a plane-bed channel (after ref.^52^) and has a narrow floodplain in areas lacking beaver (Table 1).

### Beaver cutting accumulations

Along Odell Creek we measured the total length of beaver cuttings at 90 sites, within three randomly selected 800 m stream reaches spread over 4 km (Data available at 10.17605/OSF.IO/B2JWS). The sites were selected using a stratified random sample based on 3 channel morphologic classes: point bar, straight reach, or cutbank. Within each site, a 0.5 × 0.5 m quadrat was placed at the late summer flow stream edge, as well as at 1 m and 3 m from the active channel, to measure willow cutting accumulation as a function of distance from the channel and relative elevation above low-water stage, yielding a total of 270 quadrats. Within each quadrat we summed the lengths of beaver-generated willow cuttings, as identified by clearly beaver-chewed ends, and counted the number of sprouts growing on the willow cuttings. We also recorded the surface sediment size that covered >50% of the quadrat, from silt through cobble gravel, using the Wentworth grain size classification^53^. The results for cutting accumulations along Odell Creek showed that as depositional loci, point bars were significantly more important for storing cuttings (Fig. 4 (b)), so we exclusively sampled point bars along three 800 m reaches on Red Rock Creek and five 800 m reaches on EFBD Creek

Along Odell Creek we recorded lengths of beaver generated willow cuttings within the quadrats. Using the program R^54^, we performed linear mixed effects analyses^10^ to assess parameters that may influence where and how beaver cuttings are deposited. In each model, the random effect was site name and the fixed effects were the variables of the parameter of interest. To test how each parameter affected mean willow cutting length for a site, we used Chi-squared likelihood ratio tests where the full model, including the parameter of interest, was tested against the null model without the parameter of interest.

### Beaver occupancy surveys

Walking surveys, conducted by Montana Fish, Wildlife and Parks and The University of Montana Western, and long-term monitoring on Red Rock and Odell Creeks, along with comparison of sites from National Agriculture Imagery Program (NAIP) imagery were used to document the presence and persistence of individual beaver dams. Data from all methods were used to determine beaver occupancy and abandonment periods.

### Subfossil beaver cutting accumulations

Beaver cuttings were collected for radiocarbon dating during stratigraphic investigations of streambank exposures. The beaver cuttings, generated in the stream corridor and quickly buried by progressive point bar sedimentation before decay, are not likely to have been reworked from older deposits, as uncharred woody materials previously preserved in water-saturated sediments are likely to decay rapidly when re-exposed and subjected to an oxygenated environment. The cuttings should thus reflect the age of surrounding sediments, as well as the timing of beaver activity. Rootlets were carefully removed from all wood and charcoal prior to dating. Standard pretreatment procedures^55^ were used prior to analysis. Samples were ^14^C dated using accelerator mass spectrometry (AMS)^56^ at the University of Arizona AMS facility. Three samples were sent to Beta Analytic Incorporated and were dated using radiometric methods requiring larger sample size. Radiocarbon ages were calibrated to calendar years before present (cal yr BP) using CALIB and the IntCal13 calibration curve^57^. For simplicity of discussion within the paper, individual ^14^C ages are reported using the median probability of the calibrated age distribution, providing a central point estimate (Fig. 5)^58^.

## Supplementary information

Supplementary Figure 1

## Data Availability

The datasets generated and analyzed during the current study are available in the Open Science Framework (OSF) repository at the following address: 10.17605/OSF.IO/B2JWS.
