# Supplementary material for: Beaver-generated disturbance extends beyond active dam sites to enhance stream morphodynamics and riparian plant recruitment
Source: Sci Rep. 2019 May 31;9:8124. doi: 10.1038/s41598-019-44381-2 (PMC6544642; doi:10.1038/s41598-019-44381-2)
Supplement: Supplementary file 1 — Supplementary Figure 1 [file 41598_2019_44381_MOESM1_ESM.docx]

**Beaver-generated disturbance extends beyond active dam sites to enhance stream morphodynamics and riparian plant recruitment**

Authors:

Rebekah Levine^1,2*^

Grant A. Meyer^1^

Author Affiliations:

^1^Department of Earth and Planetary Science, University of New Mexico, MSCO3-2040, 1 University of New Mexico, Albuquerque, New Mexico 87131

^2^Environmental Sciences Department, University of Montana Western, 710 S. Atlantic St., Dillon, Montana 59725

* Corresponding author: [Rebekah.Levine@umwestern.edu](mailto:Rebekah.Levine@umwestern.edu)


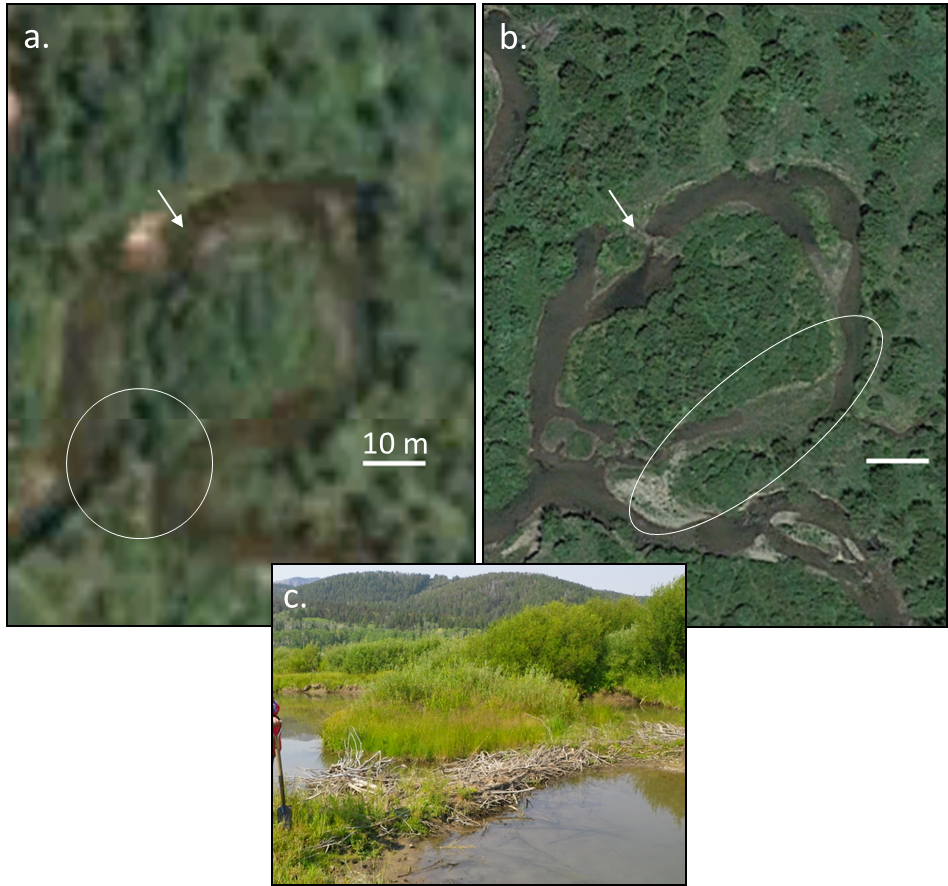


**Supplementary Figure 1.** Development of meander cutoff promoted by a beaver dam on Odell Creek; flow in all images is from right to left. **a.** Aerial view from 2005 prior to cutoff with the neck of the meander circled (map data: Google). **b.** The same site three years after the neck cutoff occurred (map data: 2014, Google), highlighting the development of the plug bar (in white ellipse) with vegetation growing on the bar. **c.** The dam site following abandonment, also indicated by white arrows in a and b. Images a and b are centered at 44°35'46.90"N, 111°47'34.38"W.
